# Diagnosis of intrahepatic cholangiocarcinoma by Raman spectroscopy provides high efficiency: A protocol for systematic review and meta-analysis

**DOI:** 10.1097/MD.0000000000023900

**Published:** 2021-01-08

**Authors:** Hongyu Jin, Man Zhang, Libin Huang, Jianqi Hao, Lingyun Zhou

**Affiliations:** a Department of Liver Surgery & Liver Transplantation Center; b Department of Gynecology and Obstetrics, West China Second University Hospital, Sichuan University, Key Laboratory of Obstetric & Gynecologic and Pediatric Disease and Birth Defects of Ministry of Education; c Department of Gastroenterology; d Department of Thoracic Surgery; e Center of Infectious Diseases, West China Hospital, Sichuan University, Chengdu, China.

**Keywords:** accuracy, intrahepatic cholangiocarcinoma, Raman spectroscopy, sensitivity, specificity

## Abstract

**Background:**

We aim to evaluate the efficiency of Raman spectroscopy (RS) in diagnosing suspected patients with intrahepatic cholangiocarcinoma (ICC), manifested by diagnostic sensitivity, specificity, and accuracy.

**Methods:**

We will research widely the articles concerning the use of RS in ICC through authenticated database including PubMed/Medline, EMBASE, Web of Science, Ovid, Web of Knowledge, Cochrane Library, and CNKI between January 2012 and November 2020, retrieving at least 1500 spectra with strict criteria. This study will be carried out in accordance to Preferred Reporting Items for Systematic Reviews and Meta-Analysis (PRISMA) guidelines. We are going to summarize the test performance using random effects models.

**Results:**

Based on the pooled sensitivity, specificity, and diagnostic accuracy, we intend to provide the relative diagnostic efficiency in ICC through RS.

**Conclusion:**

Through this systematic review and meta-analysis, we intend to provide the pooled sensitivity, specificity and diagnostic accuracy of RS in the diagnosis of suspected ICC. Other parameters like positive likelihood ratios (LR), negative LR, diagnostic odds ratio (DOR), and area under curve (AUC) of the summary receiver operating characteristics (SROC) curve will also be calculated and related figures will be drawn to help illustrate the efficacy of RS in the diagnosis of ICC.

## 1 Introduction

Following hepatocellular carcinoma (HCC), intrahepatic cholangiocarcinoma (ICC) is acknowledged as the second most common primary liver malignancy with an obviously increasing morbidity and mortality worldwide during the last decade.^[1]^ Statistically, the United States has seen an increase of ICC incidence in the last several decades, and an approximate incidence rate of 1.6 per 100,000 every year has been noted since the 21st century.^[2]^ Compared with HCC, ICC is known to have a comparatively worse long-term survival outcome, in which a satisfactory 5-year disease-free survival only possible in complete radical resections in extremely early phases.^[3]^ However, because ICC often presents with no or mild symptoms during early periods, the disease has likely developed into late stage with low possibility of a radical resection when a patient first searches clinical consultation.^[4]^ Diagnosis of ICC before surgery includes radiological diagnosis by traditional imaging techniques including ultrasound, enhanced computed tomography (CT), enhanced magnetic resonance imaging (MRI), and pathological diagnosis by a liver puncture.^[5]^ However, many traditional imaging techniques carry radiological damage and may have difficulty in judging the biological nature of the mass.^[6]^ Moreover, pathological biopsy is not normally done before a radical surgery, since liver puncture may cause uncontrollable bleeding or tumor metastasis.^[7]^ Thus, a novel diagnostic technique with high sensitivity, specificity, and testing accuracy as well as less risk should be found in order to promote early accurate diagnosis.

Raman spectroscopy (RS) was first used in chemical, physics, and biochemical industry for its ability to distinguish chemical compounds.^[8]^ In recent years, RS was applied for clinical purposes because several researches have revealed its capacity to identify the benign and malignant biological features of tumor.^[9]^ Some other studies also pointed out that RS could serve during surgery to aid surgeons by identifying the exact borderline between malignant and normal tissue.^[10]^ Meanwhile, RS examination could be carried out in vivo and compared with traditional imaging technologies, it was comparatively real-time, label-free, and non-destructive.^[11]^ Based on the physical mechanism, RS detected the change of wave-lengths or Raman shifts caused by inelastic light scattering from certain molecules inside the tissues.^[12]^ Different molecules have different combinations of Raman shifts, so tissues of different proposition were supposed to produce different spectral signatures. Therefore, distinct measured Raman spectra could provide evidence of the internal compositional features of tissues. In addition, the availability to be examined in vivo and its label-free, real-time, and non-destructive characteristics perfectly exceed the deficiencies of traditional imaging techniques and liver puncture.

Because of satisfactory diagnostic efficiency, several clinical studies deliberating the diagnostic sensitivity, specificity, and accuracy of RS in the diagnosis of ICC have been widely launched and several significant, meaningful outcomes were generated. Thus, in order to comprehensively assess the diagnostic efficiency of RS in determining the benign and malignant features of ICC which makes early diagnosis possible, we intend to carry out this systematic review and meta-analysis in order to define the clinical value of RS.

## 2 Methods

This protocol has been registered on the International Platform of Registered Systematic Review and Meta-analysis Protocols (INPLASY registration number: INPLASY2020110110; INPLASY DOI number: 10.37766/inplasy2020.11.0110. Available at: https://inplasy.com). We will document essential protocol amendments in the full review and update information in the registry. This study has been approved by the Ethics Committee of West China Hospital, Sichuan University (Chengdu, China).

### 2.1 Search strategy

Strictly conforming to the guidelines for performing meta-analysis, we intend to search extensively acknowledged authenticated databases including PubMed/Medline, Web of Science, Cochrane Library, ClinicalTirals.gov (http://www.ClinicalTrials.gov), China National Knowledge Infrastructure (CNKI) for related articles published from January 2008 to November 2020. Articles we primarily searched and identified will be subsequently screened for their quality, relevancy, and availability. No language restriction will be used. The keywords (query) of our primary search will be as follows: (((((((intrahepatic cholangiocarcinoma) OR (ICC)) OR (liver tumor)) OR (liver mass)) OR (hepatocellular mass)) AND (Raman)) OR (Raman spectroscopy)) OR (RS).

### 2.2 Article selection

Two independent reviewers will participate in the screening process to analyze the full texts and perform quality assessments and relevancy determination. Studies which meet the following criteria will be included: reporting the use of RS in ICC; being a randomized controlled trial and/or using any observational designs, including cross-sectional, case-control, and cohort designs; reporting the sensitivity, specificity values or true positive (TP), false positive (FP), true negative (TN), and false negative (FN) values, based on which sensitivity and specificity values can be calculated. Meanwhile, we will particularly exclude studies which are letters, editorials, case reports, etc. Subsequently, we intend perform a blinded cross-check to detect underlying discrepancies. If a potential discrepancy is detected, a blinded third reviewer is going to be assigned to adjudicate the conflict. The identification, inclusion, and exclusion of studies will be performed according to PRISMA guidelines.

### 2.3 Quality assessment

Quality assessment will be performed based on the Quadas-2 tool.^[13]^ In addition, the risk of bias will be obtained by RevMan 5.3 (The Cochrane Collaboration). The articles will be evaluated in the following processes: sequence generation (selection bias), allocation concealment (selection bias), blinding of participants and personnel (performance bias), blinding of outcome assessment (detection bias), incomplete outcome data (attrition bias), selective reporting (reporting bias), and others.

### 2.4 Publication bias

Publication bias will be assessed through Deeks Funnel Plot Asymmetry Test (existence of publication bias is considered positive when *P* < .05). The Deeks Funnel Plot Asymmetry Test will be conducted by Stata 14.2 (StataCorp, Texas, USA).

### 2.5 Data extraction

Two experienced investigators will independently analyze the included studies for original parameters which indicate the diagnostic efficiency as well as basic information concerning this article itself. During the process, unexpected discrepancies will be carefully discussed and resolved. More specifically, diagnostic sensitivity, specificity, accuracy, true positive (TP), true negative (TN), false positive (FP), false negative (FN) values as well as spectra values will be extracted. In addition, the title, first author, nationality, department, ethnicity, study design, sex, and median age of the patients and enrollment year will also carefully be extracted.

### 2.6 Statistical analysis

The forest plots will be generated to display sensitivity and specificity estimates using Meta-Disc version 1.4 (Clinical Biostatistics Unit, UK). The bivariate model and the hierarchical summary receiver operating characteristic (HSROC) model will be used to summarize test performance.^[13]^ We intend to use these methods to respect the binomial structure of diagnostic accuracy data, thus jointly summarizing paired measures simultaneously, for example, sensitivity and specificity or, positive and negative likelihood ratios (LRs). Meanwhile, as a random effects approach, the bivariate/HSROC meta-analysis allow pooling results in view of knowing that heterogeneity is commonplace across included studies due to different or implicit thresholds. The said approach will be carried out by metandi (meta-analysis of diagnostic accuracy using hierarchical logistic regression) command in STATA 14.2 (StataCorp, USA).

Additionally, summary receiver operator characteristics (SROC) curves will be generated to assess the relationship between sensitivity and specificity. Meanwhile, the area under curve (AUC) will be simultaneously calculated to evaluate the overall performance of RS. The SROC curved is made through Meta-Disc version 1.4 (Clinical Biostatistics Unit, UK).

## 3 Discussion

This systematic review and meta-analysis will detailedly evaluate the diagnostic efficiency of RS in ICC patients. At present, traditional imaging techniques like ultrasound, CT, and MRI (or enhanced CT and MRI) as well as a presurgical pathological biopsy are regarded as main methods to help determine the essence of liver mass before a radical surgery. However, pathological biopsy has the possibility of triggering tumor metastasis and leading to uncontrollable bleeding. Meanwhile, radiological methods sometimes have difficulty in judging the benign and malignant essence of tumor, so RS has gained increasing popularity. In other solid tumors, like esophageal cancer, kidney cancer, bladder cancer etc, RS has been proved to have comparatively satisfactory diagnostic efficiency, manifested by high sensitivity, specificity, and accuracy.^[14,15]^ Thus, through this systematic review and meta-analysis, we aim to reflect on the appliance of RS in ICC.

## Author contributions

**Conceptualization:** Hongyu Jin, Man Zhang, Lingyun Zhou, Libin Huang.

**Data analysis:** Man Zhang, Libin Huang.

**Data collection or management:** Hongyu Jin, Man Zhang.

**Data curation:** Hongyu Jin, Man Zhang, Libin Huang.

**Formal analysis:** Hongyu Jin, Man Zhang, Libin Huang, Jianqi Hao.

**Funding acquisition:** Hongyu Jin, Lingyun Zhou.

**Investigation:** Hongyu Jin, Man Zhang, Jianqi Hao, Lingyun Zhou.

**Methodology:** Hongyu Jin, Libin Huang.

**Project administration:** Hongyu Jin, Jianqi Hao.

**Resources:** Hongyu Jin, Man Zhang, Libin Huang.

**Software:** Hongyu Jin, Libin Huang.

**Supervision:** Lingyun Zhou.

**Validation:** Lingyun Zhou.

**Visualization:** Lingyun Zhou.

**Writing – original draft:** Hongyu Jin, Lingyun Zhou.

**Writing – review & editing:** Lingyun Zhou.
